# In pursuit of a better world: crop improvement and the CGIAR

**DOI:** 10.1093/jxb/erab226

**Published:** 2021-05-22

**Authors:** Jana Kholová, Milan Oldřich Urban, James Cock, Jairo Arcos, Elizabeth Arnaud, Destan Aytekin, Vania Azevedo, Andrew P Barnes, Salvatore Ceccarelli, Paul Chavarriaga, Joshua N Cobb, David Connor, Mark Cooper, Peter Craufurd, Daniel Debouck, Robert Fungo, Stefania Grando, Graeme L Hammer, Carlos E Jara, Charlie Messina, Gloria Mosquera, Eileen Nchanji, Eng Hwa Ng, Steven Prager, Sindhujan Sankaran, Michael Selvaraj, François Tardieu, Philip Thornton, Sandra P Valdes-Gutierrez, Jacob van Etten, Peter Wenzl, Yunbi Xu

**Affiliations:** 1 International Crops Research Institute for the Semi-Arid Tropics, Hyderabad-502324, India; 2 International Center for Tropical Agriculture, Km 17 Recta Cali-Palmira, CP 763537, A.A. 12 6713, Cali, Colombia; 3 HarvestPlus, Km 17 Recta Cali-Palmira, CP 763537, A.A. 12 6713, Cali, Colombia; 4 Bioversity International, Parc scientifique Agropolis II, 1990 Boulevard de la Lironde, 34397 Montpellier, France; 5 HarvestPlus, Washington, DC, USA; 6 SRUC, West Mains Road, Edinburgh EH9 3JG, UK; 7 Independent Consultant, Corso Mazzini 256, 63100 Ascoli Piceno, Italy; 8 RiceTec Inc., PO Box 1305, Alvin, TX 77512, USA; 9 Department of Agriculture and Food, The University of Melbourne, Australia; 10 Queensland Alliance for Agriculture and Food Innovation, The University of Queensland, Brisbane, Qld 4072, Australia; 11 CIMMYT, 1st floor, National Plant Breeding and Genetics Centre, NARC Research Station, Khumaltor, Lalitpur, PO Box 5186, Kathmandu, Nepal; 12 International Center for Tropical Agriculture, PO Box 6247, Kampala, Uganda; 13 School of Food Technology, Nutrition & Bio-Engineering, Makerere University, PO Box, 7062, Kampala, Uganda; 14 Independent Consultant, Hacienda Real, Torre 2, CP 760033, Cali, Colombia; 15 Corteva Agriscience, 7200 62nd Avenue, Johnston, IA 50131, USA; 16 International Center for Tropical Agriculture, African hub, Box 823-00621, Nairobi, Kenya; 17 International Maize and Wheat Improvement Center (CIMMYT); México-Veracruz, El Batán Km. 45, 56237, Mexico; 18 Department of Biological Systems Engineering, Washington State University, 1935 E. Grimes Way, PO Box 646120, Pullman, WA 99164, USA; 19 INRA Centre de Montpellier, Montpellier, Languedoc-Roussillon, France; 20 CGIAR Research Program on Climate Change, Agriculture 37 and Food Security (CCAFS), International Livestock Research Institute (ILRI), Nairobi, Kenya; 21 Institute of Crop Science, Chinese Academy of Agricultural Sciences, Beijing 100081, China; 22 International Maize and Wheat Improvement Center (CIMMYT), El Batan Texcoco 56130, Mexico; 23 CIMMYT, Mexico

**Keywords:** Agricultural policy, breeder, CGIAR, crop improvement, cultivar, food security, GxExMxS, multi-disciplinary, production

## Abstract

The CGIAR crop improvement (CI) programs, unlike commercial CI programs, which are mainly geared to profit though meeting farmers’ needs, are charged with meeting multiple objectives with target populations that include both farmers and the community at large. We compiled the opinions from >30 experts in the private and public sector on key strategies, methodologies, and activities that could the help CGIAR meet the challenges of providing farmers with improved varieties while simultaneously meeting the goals of: (i) nutrition, health, and food security; (ii) poverty reduction, livelihoods, and jobs; (iii) gender equality, youth, and inclusion; (iv) climate adaptation and mitigation; and (v) environmental health and biodiversity. We review the crop improvement processes starting with crop choice, moving through to breeding objectives, production of potential new varieties, selection, and finally adoption by farmers. The importance of multidisciplinary teams working towards common objectives is stressed as a key factor to success. The role of the distinct disciplines, actors, and their interactions throughout the process from crop choice through to adoption by farmers is discussed and illustrated.

## Teaser

This document arose from the urgent need to join forces (across nations and research disciplines) and stand together to improve the prospects for the most vulnerable population on the globe whose well-being depends on agricultural production. Here, we learn from the past, and draft future strategic guidelines that will facilitate efforts of the CGIAR institutions (https://www.cgiar.org/) to enhance the livelihoods of the less privileged through crop improvement.

**Prologue**

Long gone are the times when a single individual could encompass the evolving research across diverse disciplines. Many researchers realize that the individual human capacity, even within a single discipline, is limited and the future increasingly belongs to the establishment of efficient multidisciplinary teams that address the complexities across the research universe. Many researchers, including the authors of this paper, have observed the lack of truly effective interdisciplinary collaboration for impact in agriculture-related research (see also Cobb *et al.*, 2019; Gaffney *et al.*, 2019; Sadras *et al.*, 2020). Moreover, emerging trends in technologies, knowledge, and scientific approaches are creating new opportunities and challenges for complex strategic partnerships.

We use the lenses of a changing and evolving research context to look at the challenges particular to crop improvement (CI) which is a key pillar to reach multiple global sustainable development goals (SDGs 1, 2, 3, 5, 8, 10, 12, 13, 15, and 17; https://sdgs.un.org/goals). We look through the lens of the CGIAR research system (https://www.cgiar.org/) which represents the consortium of international research organizations aiming to reduce rural poverty, increase food security, improve human health and nutrition, and introduce sustainable management of natural resources primarily in developing countries. We called upon experts from both within and outside the CGIAR system to provide guidelines for CI teams on how to deal with the interdisciplinary complexities while reflecting the current trends in R&D of each essential discipline (original contributions are deposited in Zenodo: https://doi.org/10.5281/zenodo.4638248). From these, we provide insights on how the future strategies can build on their current proven strengths and evolve into even more effective means to reach long-term goals that are themselves continually evolving.

We reflect on the CGIAR portfolios over the decades and provide a view on how we can progress in the future to meet new challenges and the ever-changing context that embraces CI. We emphasize the CGIAR’s commitment to a pragmatic, interdisciplinary organizational model.

We stress to the readers that most of us who have been involved with the CGIAR passionately believe that it has been extraordinarily successful in meeting many of its goals and fulfilling its mission. However, we also feel that the system should not rest on its laurels and should continually improve and strive for excellence in every aspect of its work.

In the article we first present a brief overview of CI in the CGIAR and how it has evolved to meet new challenges. The main phases of CI programs are then described, with special reference to the inputs from multiple disciplines and fields of expertise. These phases start with choice of the crop to be improved and finish with the farmers growing the novel genotypes in the field and consumers using them, generally as part of their daily sustenance. Much of the knowledge and the inputs required for successful CI cut across many phases of CI programs and are dealt with as separate cross-cutting sections. We also comment on the organization of multidisciplinary teams. We do not attempt to intensively review all aspects of CI; rather we limit ourselves to areas which the group identified as critical and in need of renovation and rejuvenation, and also point to new initiatives that may be incorporated into the CGIAR’s activities in the future. Our objective is to promote discussion that will lead to CI programs that meet the CGIARs laudable goals.

Further data are available at Zenodo: https://doi.org/10.5281/zenodo.4638248.

## Introduction

Crop improvement (CI) originated when farmers, who initially selected most of the landraces, simply observed the seeds from random mating or selected ‘sports’ in vegetative crops and reproduced them to obtain improved materials (Harlan, 1972). Now, CI is a complex process fundamental for modern agriculture and a central theme in the CGIAR programs. But what is CI? Miflin (2000) recognizes that CI involves many processes and notes that the past increases in crop yields are attributable to both varietal improvement and improved agronomy. The two components, varietal improvement and crop management, complement each other: crop management often provides half to two-thirds of the yield gain (Evans, 1998). Rarely are crop yields markedly improved from a low base simply by changing the cultivar: the few exceptions to this axiom are generally associated with the removal of a major constraint such as a disease or pest through host plant resistance, as was the case with the cereal cyst nematode in Australia (Riley and McKay, 2009). A unique example is the green revolution where a major yield plateau of traditional landraces was overcome with the shortened semi-dwarf wheats and rice when planted at higher densities with added nitrogen (see, for example, Chandler, 1969; Trethowan *et al.*, 2007).

Even though CI in terms of yield generally depends on the complementary mix of cultivar improvement coupled with improved agronomy or crop management, the term ‘crop improvement’ is now frequently associated only with genetic improvement (see, for example, the draft strategy document of the ‘One CGIAR’; CGIAR, 2020). In this overview of CI in the CGIAR, we will concentrate on the narrow sense of CI as cultivar or varietal improvement but will attempt to show how it interacts with crop management. Lammerts van Bueren *et al.* (2018) proposed a systems-based approach to breeding: this is necessary as a focus just on breeding may undervalue the potential of achieving crop yield increases.

CI is an attractive option as a public good. Once a new variety is produced and distributed as a public good, the users do not have any recurring costs related to its development. Furthermore, seed technology tends to be scale neutral and farmers readily understand the idea of improved cultivars. Moreover, when host plant resistance is used as a disease or pest control measure, it minimizes the need for agrochemicals (Sharma and Ortiz, 2002). Thus, CI offers a one-off investment in research that may provide society with improved varieties that not only continue to pay off for years but also are environmentally friendly. This contrasts with many inputs associated with improved agronomy, such as fertilizers and pesticides, that farmers must continually purchase. The CGIAR, which produces public goods, has a comparative advantage in germplasm improvement and exchange, and they have become two of its mainstays (Anderson, 1998).

Cultivar or varietal improvement implies improvement in a specific trait or combination of traits for a particular purpose. Hence, as the mission of the CGIAR evolves over time, the purpose of its CI programs, the target populations and environments, the choice of crops themselves, and desired traits of the chosen crops will probably change and evolve. To help the reader understand the current and future directions of CI within the CGIAR, several of the major changes and additions in the focus and mission of CGIAR (Kramer, 2016) and how they influence CI are summarized.

In the early days of the green revolution in a world threatened by famine, agriculturalists realized that yields could not be increased without improved agronomy in the form of, *inter alia*, higher plant populations and heavier nitrogen fertilization. Breeders seized the opportunity and developed short-strawed, lodging-resistant varieties of wheat and rice that responded to higher planting densities and heavier nitrogen applications. In wheat, the high yield was combined with long-lasting rust resistance. The new varieties, when planted with traditional agronomy, gave similar or slightly greater yields than the traditional landraces. Under less favorable conditions, in the case of rice, the new semi-dwarf rice would sometimes yield less, and the marked superiority of the new high-yielding varieties was only manifested when they were intensely managed. Farmers rapidly adopted these new varieties with the necessary agronomic package, and the green revolution began. The CGIAR was born in 1972 in the euphoria of this green revolution which gave hope that hunger could be conquered (Hardin, 2008; Byerlee and Lynam, 2020) with the adage ‘To feed this world’ coined by Wortman and Cummings (1978). From its inception, the CGIAR’s clear mission was to make a sustained assault on world hunger by applying modern science and technology through centers of expertise in research and education (Hardin, 2008). This war on hunger was based on increased production largely through increased productivity as witnessed by the CGIAR mission in 1977: ‘… to support research and technology that can potentially increase food production in the food-deficit countries of the world.’ Within this framework, the decisions of those charged with CI were relatively simple. All efforts were directed to increased production, mainly through increased yield of those crops likely to increase the availability of food in food deficit areas.

The very success of the green revolution raised questions about who benefited and the effects on the environment. By the early 1980s, the mission continued to emphasize food production with the qualification that this production should be sustainable. Furthermore, both improved nutrition and the wellbeing of the poor were added to the agenda. In 1998, the emphasis was placed equally on food security and poverty eradication within the context of sustainable agricultural development and sound management of natural resources. In 2016, the overall tone of the mission transformed: ‘to advance agri-food science and innovation to enable poor people, especially poor women, to enjoy increased agricultural productivity, share in economic growth, feed themselves and their families better and conserve natural resources in the face of climate change and other threats’. Over the following years, the strategy has continued to evolve and, with the new reorganization into ‘One CGIAR’ rather than a series of independent centers, the mission is now ‘to deliver science and innovation that advance transformation of food, land, and water systems in a climate crisis’ with the vision of ‘a world with sustainable and resilient food, land, and water systems that deliver diverse, healthy, safe, sufficient, and affordable diets, and ensure improved livelihoods and greater social equality, within planetary and regional environmental boundaries’ (CGIAR, 2020).

Within this context, CI programs must also evolve to meet the expanded agenda of the CGIAR with emphasis on impact on: (i) nutrition, health, and food security; (ii) poverty reduction, livelihoods, and jobs; (iii) gender equality, youth, and inclusion; (iv) climate adaptation and mitigation; and (v) environmental health and biodiversity (CGIAR, 2020). It is no longer a simple question of producing more of a few basic staples.

## Crop choice

The first step in a CI program is selection of the crop to improve. In the private sector, plant breeders choose crops that will turn a profit for them. The CGIAR, as a quasi-public sector agency that is not primarily motivated by profit, chooses crops that enable it to fulfill its public services-oriented mission. Thus, as the mission evolves, the chosen crops would be expected to vary accordingly. With the clear mission to feed this world, rice, wheat, and to a lesser extent maize were obvious choices for the forerunners of the CGIAR. The priorities were established largely by the donors who supported international agricultural research at the time and not by the breeders (see, for example, Hardin, 2008). In the case of the International Rice Research Institute (IRRI), the decision to establish the institute was only taken after lengthy and exhaustive studies and discussions (Chandler, 1992). Later crop choices were, however, not made after extensive studies on the role these crops could play, but rather on the opinions of those who managed the individual centers and their boards of directors with the approval of the technical advisory committee (TAC). Thus, for example, in the early 1970s when the CGIAR decided which centers should work on the distinct grain legumes, the TAC noted that the need to work on these crops ‘had never been seriously questioned’ (TAC, 1974).

The expansion of the CGIAR with more centers added more cereal grains, root and tuber crops, grain legumes, and forages. In addition, two livestock centers were established. At this time, the pathway to improved nutritional level across all the centers was clearly seen through increased production, and hence availability, of a wider range of foods including more nutritious grain legumes and animal proteins.

As early as the 1969 Bellagio Conference when the foundations of the CGIAR system were established, questions were being raised about the incentives for farmers to produce more food. This concern was highlighted when Frosty Hill of the Ford Foundation was asked if traditional farmers would adopt new technologies and replied ‘Sure, if they are profitable enough.’ (Hardin, 2008). This early concern about the welfare of the farmers was, however, relegated to a secondary level of importance. The conventional wisdom that grew from the green revolution was the feasibility of both low-priced food and improved wellbeing of those that produced the food. It was claimed that the profitability of the modern farming systems was maintained despite falling real output prices due to the greater productivity (Pingali, 2007). This led to complacency and the assumption that increased productivity automatically leads to greater profitability and improved wellbeing of the farmers and farm laborers. However, deeper analysis indicated that this anodyne argument was flimsy. Farmers’ incomes in the Asian green revolution were raised by policy decisions as governments shored up credit, subsidized inputs including fertilizers, power, and water, and intervened to maintain prices (Hazel, 2009). However, with income as a rough proxy for prosperity, the CGIAR approach for alleviating rural poverty by increasing the productivity of staple food crops faster than food prices fall is risky (for more details, see Data 1 and 2 at Zenodo https://zenodo.org/record/4638248#.YOWO_i2ZOqA)).

Despite the growing evidence that it would be difficult to implement technology or production packages, as they were often called, that would satisfy the goal of reducing hunger and simultaneously improve the wellbeing of the rural poor, the CGIAR has continued to emphasize increased productivity of staple crops with a smallholder focus (see, for example, Vanlauwe *et al.*, 2014). In the early 1990s, Edward Schuh, the Head of Agriculture and World Development at the World Bank, argued that the scope of the CGIAR system could productively be expanded, noting that cash crops (and high-value crops) could generate income and employment for the rapidly growing agricultural labor force with a direct impact on rural poverty (Kramer, 2016). Pingali (2012) observed that increased productivity of staple crops could lessen the pressure on land and allow farmers to dedicate more land to the production of higher value crops. Various initiatives to produce higher value crops have been proposed in the CGIAR system, but none has been well supported and they have withered (for more information on this sad scenario, see Data 1 at Zenodo).

The CGIAR emphasizes the nutritional value of foods in its current food systems approach and recognizes the prevalence of rural poverty (CGIAR, 2020). However, the focus is on crops that are already on the agenda: no crops have been specifically selected for their potential to improve nutrition. It can of course be argued that, in terms of pro-vitamin A content, golden rice will impact many more consumers than improvement of other crops that can also provide the same micronutrient (an exception may be golden sweet potatoes). However, it can also be argued that a more varied diet would not only impact the quality and variety of the diet of the consumers but could also provide farmers with greater opportunities to increase their incomes, eat better, and generally lead more pleasant lives. The CGIAR should in the future analyze the possible benefits of pursuing multiple objectives such as improved nutrition and rural welfare by supporting improvement of crops that are not currently, or only tentatively, in its portfolio.

The CGIAR has improved livelihoods for the poor firmly established on its agenda and recognizes the prevalence of rural poverty (CGIAR, 2020). However, efforts are largely directed to the poor who purchase most of their food and not towards smallholder farmers and farmworkers. Currently, many rural people, whose predominant economic activity is related to farming, are not happy: all over the world, especially in the poorer countries, they are leaving the countryside to live in the cities, looking for a better life, especially for their children (see, for example, Saunders, 2012). Agarwal and Agrawal (2017) concluded that the multitude of unhappy farmers points to a deep malaise within agriculture, and convincingly argue that active dislike of agriculture has severe negative implications for food production and food security (for more details, see Data 1 at Zenodo). Added to this, crop choice in the current CGIAR rarely considers the role that the choice of crops could provide in making life more pleasant for the producers. For example, higher value crops, if chosen, could provide smallholders with greater incomes. Surely as an agriculturally oriented organization that is concerned with rural livelihoods, the CGIAR should purposefully choose crops that, apart from meeting other goals, wherever possible, make life on the land more pleasant!

The One CGIAR strategy brings to the forefront the attention required to ensure gender equality, opportunities for youth, and inclusion, and to promote climate adaptation and mitigation. Currently the crops chosen for improvement in the CGIAR were not chosen with a view to achieving impact in these areas. For example, whilst we are not recommending that the CGIAR work on cut flower improvement, we are aware that this industry is skewed towards providing women with more employment opportunities than men.

Often the individual CI programs attempt to satisfy a whole range of the CGIAR`s goals. This may not be an optimum strategy and it may be necessary for the new One CGIAR to choose specific crops to satisfy specific goals. Hence, as One CGIAR aligns its strategies for CI with the broadened scope of its mission, we suggest that it should review its current portfolio of crops. This review should consider the pros and cons of inclusion in the CGIAR agenda of: (i) higher value crops to provide greater income for smallholders and farm laborers; (ii) crops with high nutritional value, which may often be high-value crops, to contribute to improved nutrition and health of both the urban and the rural population; and (iii) crops, including tree crops, that may contribute to climate change mitigation. Once this assessment has been made, the CGIAR will have to find a balance between achieving the multiple outcomes it desires, paying special attention to the tensions between dual objectives, as in the case of providing the urban population with low-cost nutritious food whilst simultaneously improving the livelihoods of those that farm and produce food. As noted, this may result in the choice of specific crops to meet specific objectives, rather than attempting to achieve multiple objectives with a single crop. In this task, the foresight models now being constructed with a view to analyzing the effects of distinct hypothetical actions *ex ante* on both society as a whole as well as particular groups of individuals will play a major role (see ‘Insights into future strategies and directions’ below).

## Breeding objectives

Fixing the priorities and objectives of a CI breeding program is critical to success. If priorities and objectives are not clear and well founded, no amount of technical expertise in identifying genetic variance, crossing, and selection will make the program successful. Defining the objectives is an iterative process with constant appraisal of whether breeding is the most effective means of reaching the overall mission of the system or if, *inter alia*, crop management, improved infrastructure, and policy changes are preferable. Furthermore, breeders may not know if there is genetic variation within a particular desired trait so they must evaluate the available, useful, variation before deciding to include a particular trait. With a working knowledge of desired traits, breeders can then: (i) weigh up whether breeding is the most appropriate means to open up a new opportunity or resolve a specific problem; and (ii) evaluate negative genetic or functional trade-offs between desired traits. This all must be achieved within the scope of the target population of environments and be geared to providing benefits for the target populations and satisfaction of other stakeholders including donors.

Breeders must take a long-term view when setting their goals. From the moment the decision is made to commence a CI program to the time when a variety or cultivar is not only released, but also widely grown, is rarely less than 10 years (even for faster crops such as rice or beans). Furthermore, the commercial life of varieties can easily be another decade or more. Consequently, breeders must foresee what the growers and consumers will need in 10–20 years time: *inter alia*, consumer preferences may change; climate and weather patterns are likely to be distinct; new diseases and pests may appear; farm labor may become scarce and more expensive; agricultural and food policies may change; and certification and regulation of agricultural products will probably become more onerous. CI teams require inputs from multiple disciplines, allowing them to glimpse into the future (see ‘Foresight models’ below).

An early example of looking ahead within the CGIAR system was that of cassava at CIAT in the early 1980s. There was no question of the production potential of the crop, but rather on the demand for what some considered an inferior good with very inelastic demand and, therefore, with limited growth prospects. The program was put on hold until a series of demand studies confirmed that there was a large expanding market for cassava, particularly in south-east Asia. The CI efforts were reoriented to emphasize south-east Asia with a clear objective of alleviating rural poverty through improved cassava production technology (Lynam and Janssen, 1992; Kawano and Cock, 2005).

Private sector breeders develop a product profile of the variety that farmers would prefer relative to those they are already growing and then use this to define the breeding objectives (Cobb *et al.*, 2019). A product profile is roughly defined as a set of targeted attributes that a new plant variety should have in order to be successfully adopted by farmers. A product profile focuses breeding efforts on the key traits that drive incremental value creation (Cobb *et al.*, 2019). Despite significant investment to help public sector breeders understand the importance of product profiles (e.g. https://plantbreedingassessment.org), many public sector breeders still have not adopted this concept. Sometimes the profiles can be simple and hence the breeding objectives are also simple yet sufficient to deliver impact. Growers of Andean Blackberry (*Rubus glaucus*) dislike pruning and harvesting the thorny blackberries. A thornless mutation was discovered and selected, and—even though it yields no more than the thorny varieties—it is now widely adopted in Ecuador and Colombia by farmers (Portalfruticola, 2014). However, in the CGIAR, most of the CI programs are charged with meeting multiple objectives including skewing of benefits to a target population of the underprivileged; consequently, developing product profiles and fixing breeding objectives is almost never simple. Furthermore, the CI programs generally are expected to cover a wide range of environmental conditions and possible management options. At the same time, to spread the costs of breeding for this wide range of conditions, breeders are forced to look for varieties capable of performing well over a wide range of conditions (see Data 10 at Zenodo). This leads to what we denote as the ‘Breeders dilemma’. The farmers want a variety that performs well on their farm, whereas the breeder aims for a variety that functions well over as wide an area with as many adopters as possible. These conflicting demands may lead to the breeder having to find a delicate balance between what is best for one single farm and broad adaptability.

The green revolution was characterized by broadly adapted varieties that were grown around the world, with the environments stabilized by irrigation and fertilizers, and, in some cases, pesticides. Later, especially with non-irrigated crops, recommendation domains, defined as a group of roughly homogeneous famers with similar circumstances for which we can make more or less the same recommendation (Byerlee *et al.*, 1980), were used, with breeders developing specific materials for the distinct recommendation domains. We note that this concept developed in the 1970s had a strong social component, with the emphasis on farmers, not farms. The emphasis on social aspects is currently re-gaining importance. Many options are now open for breeders to define and characterize these recommendation domains or target environments (see ‘Target population environments’, and Data 3, 7, and 12 at Zenodo).

Unlike commercial CI programs, with the farmer as the only major client or customer, the CGIAR programs must satisfy not only farmers’ needs but also more diffuse objectives on nutritional status, rural prosperity, and inequality, with emphasis on gender inequality, natural resources, and the environment, all in a world of uncertainty due to climate change. The foresight models and identification and characterization of target populations (see Data 12 at Zenodo) help the breeders identify potential traits that could be incorporated into new varieties so that their adoption by farmers would meet the overall goals.

As breeding teams decide on priorities, they must be aware that each added trait will, other things being equal, reduce the potential genetic gain through an influence on selection pressure. Hence, wherever possible, breeding objectives should be limited to a few critical priority traits. The first step is to determine whether useful genetic variation exists for the desired trait within the available germplasm of the crop species itself or whether there is the possibility of incorporation from other species or through use of techniques such as gene editing (see Data 2 at Zenodo). Obviously, if there is no genetic variation nor the likelihood of creating it within the crop in question, then the trait is eliminated from the breeding objectives. This process may still leave many traits as optional breeding objectives. The breeders should then evaluate whether breeding is the most effective means of meeting a particular goal. For example, biological control may be more effective as part of a pest management program than host plant resistance, or use of herbicides for weed control may be more effective than developing varieties that compete well with weeds.

Determination of breeding objectives is further complicated by trade-offs between desirable traits. Thus, for example, there may be a trade-off between some of the traits that provide yield advantage in a particular drought context but reduce yield under well-watered conditions (e.g. Kholová *et al.*, 2014; Cock and Connor, 2021). These trade-offs are often manifested as negative genetic correlations and must be considered when fixing priorities. Targeted methodologies for trait genetic mapping such as genome-wide association studies (GWAS) can help breeders assess the genetic basis of any trade-offs and improve the design of selection methodologies.

CI, by its very name, suggests that the performance of the crop will be improved. However, the Red Queen effect of ‘it takes all the running you can do to keep in the same place’ is a frequent feature of CI. Breeders continuously incorporate new traits into their programs, as new pests and diseases or new strains appear, just to maintain yield levels. Intensive cropping systems may also lead to deterioration in the environment, such as salinization resulting from over-reliance on irrigation. Under such circumstances, breeders often struggle just to maintain yields. The importance of this maintenance breeding can easily be lost if breeders are increasingly expected to provide proof of genetic gain as the hallmark of success (see ‘Genetic gain and the breeder’s equation’ below).

**Fig. 1.**
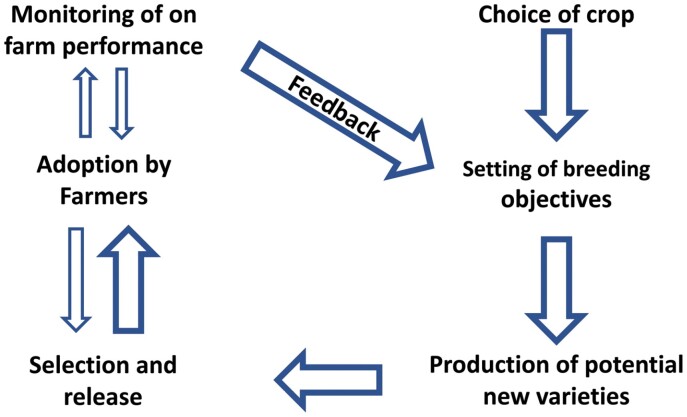
The schematic overview of the manuscript logics and structure. It illustrates the main processes involved in successful conceiving, creating, releasing, and adoption of new crop technologies.

Priority setting is an iterative process that requires a deep understanding of the crop production processes and the whole supply chain to the final consumer, which can only be obtained from a multidisciplinary approach (the details of relevant disciplines and their contribution to breeding are visualized in Fig. 1, and in Figs S1, S2, and Data 2–19 at Zenodo).

Product profiles of a particular crop are dynamic and will change with time. This is well illustrated for tropical rice within the CGIAR. The original green revolution IR8 rice variety was of poor grain quality and susceptible to several important diseases and pests; the next stage of breeding produced IR20 and IR26 with high yield potential and better disease resistance; later IR64 added grain quality; and now, with golden rice, improved nutritional quality.

## Production of potential new varieties

Through cyclical breeding programs, breeders aim to combine desirable traits and alleles in single genotypes. In this process, tens of thousands of novel genotypes are produced. Only a few will meet all the product profile criteria, be selected, and eventually grown by farmers. Nowadays, breeders have many options for combining these desirable traits to create new varieties (Fig. 1). Depending on the crop, product profile, and budget, they can choose from methodologies that range from traditional crossing methodologies to incorporating exotic genes from close/distant relatives (for more details, see Data 2 and 3 at Zenodo).

The traditional source of genetic variation is from germplasm collections. One of the major strengths of the CGIAR CI programs has been and continues to be the large germplasm collections of several crops (see Data 7 at Zenodo). Germplasm collections are of little use to breeders if they are not characterized. CI programs now have many tools to characterize and select suitable material for their crossing schemes. For example, an effective proxy for a target trait/phenotype in the form of an underlying gene/allele/allelic combination can facilitate the work of breeders. However, to use these tools, a knowledge of genetics/genomics is indispensable. The increased accessibility and cost-effectiveness of molecular tools open the way for CI teams to identify genes associated with desirable traits or combinations of these traits.

Breeders can and do systematically mine genes and gene combinations (e.g. GWAS, genomic selection-based approaches) from diverse populations. In addition, breeders now have the option of targeted manipulation of specific genes. Editing single genes, whilst leaving the rest of the genome unedited, opens up a whole new range of opportunities for breeders. The flourishing genome manipulation methods now available are already used by many programs (Data 2 at Zenodo). Several CGIAR centers (CIMMYT, IRRI, IITA, and CIAT) have produced genetically edited crops (GEds) as tools for breeding. Consumers still may perceive the potential dangers and do not understand the benefits of genetically modified organisms (GMOs) and GEds. However, regulators in many countries understand the advantages of both GMOs and GEds, and the CGIAR breeders should actively work to make suitable GMOs and GEds available to those countries interested in and willing to accept them. Simultaneously, scientists who use genome editing or produce GMOs will need to communicate effectively with consumers and regulators to increase public awareness of the potential benefits and to allay fears of potential dangers. Moreover, the public and regulating agencies should be informed of the distinction between GMOs and GEds, the latter of which are much more readily accepted and approved.

Irrespective of the way in which the genotype is manipulated in CI, it is the expression of the genotype—the phenotype—that determines the success of the outcomes of selection. Therefore, where the genetics of the trait are not known or are exceedingly complex for dissection, the identification of suitable material for use in CI relies on phenotyping. Phenotyping is still a significant bottleneck in CI programs. Due to rapid technological advancement penetrating the realm of all biological disciplines, new high-throughput phenotyping methodologies are likely to become readily available in the coming decades (Data 4 and 5 at Zenodo) and offer the opportunity to characterize and choose germplasm rapidly and at a lower cost than with traditional phenotyping.

It is the phenotype in the context of a farmer’s field that will finally determine the farmer’s decision to adopt the variety. Therefore, CI teams must always keep in mind that a major difficulty with genotyping and phenotyping methodologies is that the genetic determination (G) of phenotype often depends on the environment (E) with a G×E interaction. Hence, as the new methodologies for genotyping and phenotyping become available, CI programs must improve their knowledge and understanding of these G×E interactions.

## Selection and release

Breeders select and advance a small number of genotypes from the tens of thousands of novel genotypes that are normally produced in successful breeding programs (Fig. 1). Initial testing often includes elimination of those materials with obvious defects, such as susceptibility to an important disease or lodging, or an undesirable plant type. Some materials may be eliminated without even field testing using a range of high-throughput techniques for testing seedlings such as through use of validated genetic markers (for more details, see Data 3 and 4 at Zenodo). Once materials with evident defects have been eliminated, plants are field tested under conditions representative of target environment(s). At this stage, genotypes are preferably tested as plant communities so that their ability to produce with interplant competition is evaluated. Multilocation trials are often used to evaluate G×E; however, a standard management or technology package is generally applied, as selection for stability over a range of management (M) or analysis of G×M in the early stages of selection has been excessively challenging. It should be noted that breeders have frequently chosen levels of management in the selection process that are in accordance with what farmers can be expected to achieve. Thus, in several CGIAR CI programs, selections was under low purchased input management levels (see, for example, Lynam and Byerlee, 2017). However, opportunities for realizing the crop potential through M may be missed when only one standard management scheme, often based on what is optimum for the currently grown cultivars, is used. Progress with *in silico* crop simulation methods may close this gap and allow CI programs to identify phenotypes well adapted to specific management practices. Even if the crop management (M) is considered, the CI programs need to understand the social milieu (S) which varies between farms. However, the management will probably reflect the social milieu and, hence, if CI programs understand this M×S interaction and consider the M component, they can embrace the overall G×E×M×S (GEMS) continuum (see Data 2 and 12 at Zenodo).

The current limited use of on-farm trials in the selection process by the CGIAR and NARS in developing countries is in stark contrast to prevailing practices of large private seed companies in developed countries, which, with larger budgets and many collaborating growers, may cover thousands of farms every season for each crop and region, to provide detailed insights not only into G×E (Marko *et al.*, 2017) but also into GEMS. While the CGIAR has emphasized G×E, there is scope for a better understanding of M and S. In Nigeria, farmer selections of cassava (often escapes from yield trials or progeny from chance seedlings in the field) that had not been released as varieties made up more than half of the area under modern varieties (Thiele *et al.*, 2020). Furthermore, Thiele *et al.* (2020) confirmed their hypothesis that breeders give insufficient priority to consumer-preferred traits, which are difficult to assess, for roots, tubers, and bananas. Thus, breeders may not be selecting materials with all the traits desired by farmers, as witnessed by the escapes, but this was not for lack of genetic variation in the desired traits. At the same time, it should be noted that several of the traits of the modern varieties—high productivity and disease resistance—were almost certainly present in the escapes, indicating that the breeders had made a useful contribution by introducing genetic materials with these characteristics. The selection of escapes illustrated by Nigerian farmers is a non-formalized case of participatory variety selection (PVS; Data 14 at Zenodo). In PVS, farmers participate in the testing and selection of experimental varieties (Ceccarelli and Grando, 2020; see Data 14 at Zenodo). PVS, whether formalized or informal, is an attractive option, particularly when traditional selection methods may discard varieties that farmers consider desirable in their social and physical environment. More generally, detailed, relevant, feedback from farmers’ fields would help breeders select varieties which farmers like and would adopt while providing inputs for constant refining and evolution of the product profiles for the future. There is no doubt that working directly with the farmers fosters valuable knowledge exchange between farmers, breeders, and others (for more details, see Data 13 and 14 at Zenodo).

Selection for climate resilience is difficult as it may be impossible to find an environment that emulates future scenarios and socio-economic conditions. Here, the role of crop modeling tools becomes indispensable as these can expand the GEMS analysis across space and time to include anticipated future scenarios including climates. Crop models encapsulate how plants respond to the environment and can play a critical role in the design of target phenotypes (Messina *et al.*, 2011; Cooper *et al.*, 2014; Vadez *et al.*, 2015; Bustos-Korts *et al.*, 2019). This framework is used in modern CI to improve the understanding of production environments and for efficient design of a product suited to these production contexts. We also suggest that these technologies may facilitate *ex ante* evaluation of genotypes for performance in a wide range of distinct socio-economic and physical environments characteristic of smallholder agriculture without the need for massive field testing. The modeling approaches can already integrate a few components of the socio-economic dimension, but these still require much refinement and more data to support their development, particularly when related to consumer preferences that are often subjective. Nevertheless, the CGIAR would be well advised to invest more in crop models that not only consider the variety, soil, and weather continuum, but also include management and social preferences.

## Release and adoption of new varieties

It is widely recognized that adoption has been and still is a major problem (see, for example, Alary *et al.*, 2020). A common feature running through the data available at Zenodo is the large number of varieties from CGIAR programs that have never been adopted by farmers, often due to a poor definition of stakeholders’ requirements in the product profile. Thiele *et al.* (2020), with reference to root and tuber crops, observed poor adoption when consumer preferences were not considered (note: we use consumer in a broad sense to include not only the farmer but also other value chain actors, such as processors and market intermediaries). This consensus among the authors of a large number of varieties never being adopted is sadly not reflected in the literature as breeders are unlikely to write about their failures, and journals are equally reticent to publish them. Nevertheless, despite the large number of successful examples of CI, we feel that attention should also be directed to analyzing those cases where varieties were not adopted by farmers, to understand why, with a view to improving the effectiveness of CI programs.

Within the CGIAR system, the challenge of achieving adoption is tortuous. In many cases, the CGIAR breeders see their role as pre-breeding of materials which others in national programs will use as the basis for development of varieties. In other cases, the CGIAR may send segregating populations or a range of clones for final selection by local programs. An attractive feature of this approach is that local programs select materials that meet the exigencies of the local environment and preferences. Nevertheless, these advantages are counterbalanced by the CGIAR breeders not being in direct contact with the users of the selected varieties, resulting in no direct feedback on deficiencies in the materials produced. Furthermore, if varieties are not adopted, the CGIAR breeders are not directly accountable for the failure in the overall system. In the early days of the CGIAR, in-service training programs for national program staff were an integral feature of many of the CI programs. The CGIAR-wide evaluation of training in 2006 indicated that the CGIAR should continue training compatible with their research priorities and develop strategies to do so in ways that strengthen NARS capacities (Stern *et al.*, 2006). However, it was noted that this was becoming more difficult with the move towards shorter term project funding. In several cases, CGIAR breeders have been outposted to work closely with national program staff in the development of new varieties. We suggest that breeding programs must ensure continuity of efforts from pre-breeding within the CGIAR through selection and delivery to farmers by the NARS or other entities. This may be achieved by various means, including a renewed emphasis on training of national program staff coupled with outposting staff, both of which help establish the long-term partnerships required. Furthermore, the donors could consider supporting networks that include activities not only in the CGIAR itself, but also in the local and national programs (see, for example, Nestel and Cock, 1976)

Many breeding programs go through the process of multilocational trials managed by scientists followed by rigorous analysis of the data and formal release of varieties. Participatory plant breeding (PPB) and PVS, which frequently do not follow the multilocational, scientist-managed trials, can lead to high rates of adoption, although questions have been raised about the cost. Mangione *et al.* (2006) and Ceccarelli *et al.* (2012) argue that the higher rate of adoption more than compensates for the high cost: the cost of producing varieties that nobody grows in conventional public breeding programs is immense.

Robust methodologies on how to standardize the PPB and PVS approaches for the myriad social and physical environments occupied by smallholder farmers are just emerging (e.g. Bentley, 1994; van de Fliert *et al.*, 2002; Dhehibi *et al.*, 2020). These methodologies are especially pertinent with respect to PPB, where the cost/benefit of breeding for the myriad distinct conditions of smallholder farmers has to be carefully weighted.

The Colombian sugar industry has developed a rigorous variation on the theme of PVS. The privately funded National Sugarcane Research Centre (Cenicaña) monitors every harvesting event of sugarcane in the main sugarcane-growing area with details of the environmental conditions, soils, various management practices, variety, and, crucially, yield and quality (Cock *et al.*, 2011). Cenicaña does not officially release its varieties: field trials are established with a range of varieties and the mills or farmers select those they like and plant them commercially on a small scale. Data from the monitoring system are made available to growers on a web-based page where they can compare the new varieties with their current materials under well-characterized conditions and decide which to continue planting. This system allows growers to select the promising varieties they prefer and provides them with data to decide which varieties to multiply and grow on a larger area, with no official varietal release. Similarly, in several cases of PVS (see Data 14 at Zenodo) and of escapes that are adopted by farmers (see ‘Insights into future strategies and directions’ below) there is no official release of the varieties. Farmers simply adopt what they like.

There are recognized risks associated with systems based on farmer selection without rigorous testing. For example, farmers may plant disease-susceptible varieties which do well on isolated fields but are disastrous when grown on a larger area. Thus, a careful balance is required between letting farmers grow what they choose and careful monitoring of field performance to provide them with feedback on both the advantages and dangers of growing specific varieties. Currently many of the crops jointly developed by the CGIAR and national programs maintain rigid processes passing through multilocational trials and formal release programs. We suggest that for some crops and circumstances, other approaches that permit more active participation of farmers in the selection process and the decision of what to grow should be explored to either replace or complement the more formal processes. Within this framework, monitoring of the performance of farmer-selected materials is required to ensure rigorous evaluation of suitable management and growing conditions for these novel selections.

When new varieties are grown, management practices frequently must be adjusted. It is not realistic for the CGIAR to adjust management practices to each of the varieties that come out of its pipeline. However, monitoring information on commercial fields may be used to predict responses to variation in E (largely soil and weather) and a whole range of management practices (M) (see, for example, Jiménez *et al.*, 2016). Thus, for example, nutrient response curves can be generated from production practice and yield data obtained from farmers’ fields (e.g. Palmas and Chamberlin, 2020).

Monitoring of farmer experiences leads to the possibility of variety selection based on digitally supported large-scale participatory research using the principles of crowdsourcing and citizen science (van Etten *et al.*, 2019a) and how this could be done by linking georeferenced trial data to daily temperature and rainfall data (van Etten *et al.*, 2019b). Continued statistical innovation has helped to support more flexible on-farm trial formats (van Frank *et al.*, 2019; Turner *et al.*, 2020), and it is now possible to analyze G×E interactions from data obtained on farms. While big data approaches are a daily chore in most CI programs, the massive amounts of data produced by farmers themselves have hardly been touched. If the CGIAR were to mount systems of crop monitoring in conjunction with partners in national or local programs, it would obtain feedback on what farmers need, how varieties perform, and adjustments needed to management practices. The monitoring would also provide farmers with information on which variety is most suitable for their farm and how to manage the chosen variety. Furthermore, it would provide early warning of potential problems with a new variety and is of particular importance to provide farmers with information on varieties coming from PVS programs.

## Insights into future strategies and directions

Since most of the contributors have had experience working closely with the CGIAR system, they have brought an invaluable, practical insight into the system’s operation. In the following subsections, we discuss the emergent themes of opportunities and challenges faced by CI in the CGIAR. The generic framework for CI which we use to distill the points of view highlighted by the experts is shown in Fig. 1. We encourage readers to consult with the data available at Zenodo (https://doi.org/10.5281/zenodo.4638248) which provide greater detail and more examples to support the suggested paths that CI should take in the future.

### Continuity

In their classic paper ‘Slow magic’, Pardey *et al.* (2001) stress the importance of continuity in CI programs. The current CGIAR structure based on short-term projects is incompatible with this appraisal. Synchronization and dialog between the donor agencies (Gaffney *et al.*, 2019; Sadras *et al.*, 2020), national-level research institution, and policy makers, together with the top management of CGIAR institutions is required to break out of the short-term project systems and return to longer term support.

Monitoring and evaluation of successful CI programs provides evidence of the high pay offs from CI (Byerlee and Moya, 1993). These high pay offs may induce donors to invest in longer term programs. However, most evaluations have concentrated on productivity gains and only recently have begun to consider impacts related to other goals such as facing the challenge of climate change (Barnes, 2002; Dantsis *et al.*, 2010; Alston *et al.*, 2020). Quantifying the social and economic impact from CI investments (Alston *et al.*, 2000, 2020) both *ex post* and *ex ante* is of vital importance for ensuring that donors and other agencies see the high benefit to cost ratio of investment in CI and hence continue or, preferably, increase their commitment to longer term funding. This funding should look at the whole continuum from defining product profiles of individual crops, to ensuring that local or national programs that receive materials from One CGIAR have the resources needed to make sure that farmers obtain improved varieties.

Guaranteed long-term support is, however, not without risks. Complacency and a lack of accountability may creep into programs unless they are periodically held to account, as was the case in the early days of the CGIAR, with periodic intensive internal and external reviews of progress of the individual centers and their programs.

### Foresight models

Foresight models now help the CGIAR decide on where and how agriculture is heading and its role in the future and how to put long-term breeding goals into this context (for more details, see Data 19 at Zenodo). Foresight is, by its very nature, a science of integration. The strength of strategic foresight is that it does, indeed, allow for systematic exploration of alternative futures integrating disciplines from climate science and crop science to economics, spatial analysis, and, critically, CI to co-create robust and relevant foresight analyses. The incorporation of various modeling approaches into strategic foresight can help overcome a lack of adequate observations from field trials and, simultaneously, quantify crop response under future climates and different spatially explicit conditions *in silico*, while also considering future demand and other socio-economic or policy factors (Kruseman *et al.*, 2020). Foresight helps us to gain a targeted understanding about both the sensitivity of different investment strategies in CI to exogenous factors such as population growth, changes in income, and climate change (Ceccarelli and Grando, 2020), and even different investments in agronomic strategy such as sustainable intensification (Rosegrant *et al.*, 2014). Given the need to accelerate advances in breeding-based adaptation strategies, quantitative foresight models are viable tools that can be used to understand potential implications of different breeding strategies. Quantitative foresight models will help us understand not only how intended improvements might affect crop performance in a spatially and temporally explicit manner (Kruseman *et al.*, 2020), but also how these changes respond to (and affect) future biophysical and socio-economic conditions (Wiebe *et al.*, 2018). Thus, strategic foresight provides a means to systematically explore alternative futures. Integration of foresight into a CI program will provide integrative ‘futures evidence’ that serves as a decision support tool that complements traditional priority-setting methods and expert knowledge (see Data 16 at Zenodo).

Crop foresight models can provide a more solid basis for strategic decisions such as future crop choices. However, the futures that foresight models anticipate may inject present bias into our futures perspective; if economic outcomes are seen as the main priority, foresight models will, predictably, suggest investment in CI that targets many of the most widely consumed commodities (Wiebe *et al.*, 2020). On the other hand, if the desired future is related to achieving a particular environmental outcome, such as eating within planetary boundaries, or a rural welfare objective, the corresponding strategies can be substantively different (Springmann *et al.*, 2018). The appearance of more nuanced and niche markets which may lead to a more diversified diet and potentially increased rural incomes will need to be incorporated into the foresight models. Furthermore, understanding the prerequisites for adoption of new technologies including CI along the value chains will be an important driver for directing effort in CI.

Applying choice experiments using simple games, which trade-off a series of alternative traits or business choices, would allow us to understand the primary motivations of end users and hence which traits should be included in CI strategies (e.g. Naico *et al.*, 2010; Claessens *et al.*, 2012; Aravindakshan *et al.*, 2021). Agricultural economic studies can be augmented by real-time market data to support forecasts for desired traits for CI programs. This allows flexibility for understanding the risks from different CI strategies and can assist breeders to respond to market signals or policy interventions in the development of varieties that will be required as changing preferences of end users and policies alter market demand. Economists, working with CI scientists, can therefore integrate and inform a more dynamic understanding of traits and how future scenarios will affect present crop adoption trajectories (see Data 15 at Zenodo).

### Breeding teams

CI or breeding teams should be formed to determine how the long-term opportunities for change identified by the foresight models can be realized. Traditionally the breeders decided on the desired traits, made crosses based on what they felt would be likely to combine these traits in the progeny, and then walked plots and visually selected individuals based on desired characteristics, their visible correlates, personal whims, and known (often memorized) pedigree information. Progressively, with more systematized and centralized CI programs, the romantic notion of an individual breeder exercising art through observation and careful note taking has shifted to a cross-disciplinary data-driven science that is more evidence based. While this transition began more than a century ago, it still may be emotionally disturbing for some breeders who make educated guesses in a data-poor environment. Progressive breeders who understand that all breeding is subject to the pitfalls of approximating veracity and search for reliable data sources, while still subject to systematic bias, are less subject to the prejudices of human perception. Modern CI is a skill, on the one hand informed by aspects of biology, chemistry, and mathematics, and the environment in which the crop is to be grown, and on the other hand driven by the needs of farmers and the consumers of their products. Fundamentally, a breeder’s job is to integrate all this information and to identify varieties or cultivars suitable for the biophysical and socio-economic environment of interest.

Much of the success of the CGIAR CI programs can be attributed to the establishment of multidisciplinary CI teams. Nickel (1983) indicated that CI was best accomplished by organizing the research scientists into interdisciplinary programs or teams along [crop] product profiles. Modern breeding programs are complex, and one individual cannot gather, organize, interpret, and summarize all the required information from multiple sources. Breeding teams need to implement modern information ecosystems or platforms based on data management and decision-support software that integrate information and apply sophisticated analytical workflows to the information. These platforms facilitate setting of breeding objectives and strategies and transparent design of breeding operations based on a whole array of key competences and areas of expertise from within the team. Although discussions and contributions from the whole team provide the guidelines, the team leader is responsible for taking well-informed, critical, decisions and for providing ‘clarity of direction’ for the team. In this participatory mode of making decisions, clearly those that do not buy into the agreed directions or are not able to fulfill their role have no place in the team.

The team lead must weigh the disciplinary contributions according to both the breeding objectives and the funding available. The leader should be wary of attempting to deliver on many targets with a consequently high probability of failing to meet any of them satisfactorily. It is better to ensure delivery of a few well-defined and achievable objectives than to partially fulfill a large wish list. Hence, despite the generic call for integration and multidisciplinary approaches, paradoxically, breeding needs only as many contributions from other disciplines as is required to meet the breeding objectives and deliver on the target product profiles.

Once a crop and the breeding targets have been chosen, breeding becomes a major exercise in logistics. The breeders need to organize, just to mention a few: product profiles; access to genetic variation principally from germplasm banks; facilities for phenotyping and genotyping; determination of the target population of environments (TPE); development of selection protocols including identification of selection sites and crop management practices to be used within the TPEs; data management systems; and close relationships with partners. The logistics must be organized within the restrictions of available expertise and financial support.

The leader of CI programs, apart from having a high level of technical competence, should be an excellent manager and leader, with interpersonal, strategic, and tactical skills. The CI-lead must create an atmosphere of trust not only for sharing the successes but also to realize that these are built on many failures, which require team resilience to overcome. Breeders who neither listen to the comments of other breeders nor have close contact with farmers are rarely successful. Simultaneously, the leader must be aware of advances in science in a range of disciplines so that the program can seize new opportunities and innovate. The CI-lead should lead by example and ensure, both personally and for the team, the highest quality in all aspects of the program. There is today a real concern that breeders will spend too much time staring at computer screens and not sufficient time understanding the intricacies of plants and their relationship with other organisms, overseeing the critical field selection processes and comprehending the day-to-day problems of farming. An important function of the team leader (frequently overlooked in the CGIAR) is to ensure that incentives to members of the breeding team are linked to the overall breeding objectives. John Nickel on his first day as the new director of CIAT in 1973 reminded the scientists present that as CGIAR employees our success is measured by the rate of improvement in livelihoods of those less fortunate than us and not by the number of academic papers we produced. Unfortunately, these wise words are often unheeded (see, for example, Cobb *et al.*, 2019), and many scientists in the system feel that excessive weight is given to publications in the evaluation procedures.

Breeders are used to failure: of the progeny they produce, only one in many thousands of genotypes eventually becomes a successful variety. Breeders can, however, learn from negative results. When they rogue out materials that are not promising, they learn which parents are more or less likely to produce potential useful progeny. Moreover, often important negative results, such as allelic variation in ‘x’ that does not contribute to heritable variation in ‘y’, are neither published nor systematically recorded. Nevertheless, these relationships gained through the experience of individual researchers contribute to their understanding of the issue in question. A multidisciplinary team needs to integrate such experience and understanding, and that can only be by open and unfettered communication. This means firstly that individuals should be encouraged to ask challenging questions or to be able to pose them in a way that reveals a lack of knowledge or understanding, and secondly people should not be ashamed of apparently negative results and should openly discuss them and their significance for the program. Thus, for example, if no genetic variance is found for a desirable trait, this should be openly discussed so that a decision can be made as to whether to continue searching, or to decide on an alternative strategy, or not to work further with improvement of that trait. It is not the researcher’s fault if there is no genetic variation! The general management principle that looking for the guilty person in the team to blame when things do not work out as expected is extremely unproductive and detrimental in CI programs.

The ‘culture’ of a team or organization reflects how its collective experience, knowledge, and understanding are maintained, nurtured, and deployed, and this is of special relevance to succession planning. The quality of the succession planning ultimately reveals the priorities of an organization. One of us, who has been involved in both animal improvement and CI programs, has always insisted that there should be a backup leader of the breeding program who can step into the leader’s shoes if, for any reason, the leader leaves the program or is no longer able to carry out their duties. This redundancy is increasingly difficult to achieve in the present, cost-conscious, cost-recovery, project-based, environment of the CGIAR. However, it is vital to ensure the continuity of CI programs that cannot simply be switched on or off.

### Genetic gain and the breeder’s equation

The breeder’s equation provides a framework for determining the rate of progress of breeding and contributing components in terms of genetic gain (Equation 1).


$$\begin{array}{l} \;\Delta \;G_{year}=\frac{i\;r_{AI}\sigma_{A}}{L} \end{array}$$ (1)

where *i*=selection intensity, γ _*AI*_=accuracy, σ _*A*_=genetic standard deviation, and *L*=generation interval.

The genetic gain for target traits is measured as a function of the selection intensity, accuracy of selection, the magnitude of genetic variance (measured as the SD of the trait in a reference population), and the interval between cycles of the breeding program. It is important to understand that the breeder’s equation is not a single trait equation with *G* being solely yield. *G* has to be understood as a multivariate, multitrait, framework. Multidisciplinary teams are better equipped to decipher this multivariate complexity.

There are frequent trade-offs between advances in one trait and another. These trade-offs may sometimes be manifested as negative genetic correlations between traits, such that positive genetic gain in one trait is associated with negative genetic gain in the other. In some cases, it may not be possible for the negative genetic correlation to be broken. However, if there is a high metabolic cost to providing pest resistance, the negative relationship between yield in the absence of the pest and resistance will be difficult or impossible to break. Furthermore, *ceteris paribus*, as the number of traits is increased, the potential rate of progress in each trait decreases.

As breeders strive to achieve genetic gain, the plant breeding paradigm is changing from selection of phenotypes toward selection of genes. Plant breeders bring together in one genotype many alleles that maximize the expression of the desired traits of the product profile (Koornneef and Stam, 2001). However, because genes do not function as single entities, it is necessary to know how numerous genes function together. Passioura (2020) eloquently discusses how translational research involves gaining knowledge that flows from the level of the gene, through metabolites, membranes, organelles, cells, tissue, organs, plants, and communities. This process of understanding the distinct levels as one moves along this progression should allow CI teams to envisage the expected effects of changes at the gene level on the overall development of the crop. However, sometimes CI teams tend to short-cut the process, jumping directly from the gene to the plant or community level. This approach is appealing as it may reduce the time needed to progress. However, it is dangerous, as a lack of understanding of how the individual components interact and make up the whole continuum can lead to looking for silver bullets that use one or very few genes to solve a problem, without understanding how that one gene interacts with all the others. Hence, breeders may select genes apparently associated with a particular trait only to find that it does not produce the expected results in a given context (Tardieu *et al.*, 2018; see, for an example, Data 1 at Zenodo).

Basic research, perhaps led by universities and academia in general, that provides insights into the development processes of plants and their response to changes in the environment would reduce the temptation to look for silver bullets. Furthermore, a more profound understanding of how plants function would reduce reliance on black box approaches to construct crop simulation models that are becoming increasingly important in determining *ex ante* how distinct traits will interact in the whole plant environment.

The current emphasis of the CGIAR system on quantifying genetic gain in farmers’ fields becomes fraught when concepts such as maintenance breeding are introduced into the equation. However, it is not only with maintenance breeding that genetic gain is difficult to quantify. For example, how do you quantify genetic gain of multiple traits such as yield, combined with yield stability and improvement of nutritional quality? How much of yield gain in farmers’ fields do you apportion to genetic gain and how much to the improved management and even increased carbon dioxide concentration in the environment? The improvement of nutrient densities in staple crops for a better fed world should evidently continue. Nevertheless, how do you meaningfully define the genetic gain of nutrient status at the field level? Similarly, if a variety is developed that makes life more pleasant for women, how do you measure the genetic gain? Although it is intellectually appealing to have a single figure of annual genetic gain at the farmer’s field, this figure will depend on some heroic and subjective assumptions in its estimation. Hence, while it is attractive to have a simple, single quantitative measure of progress such as genetic gain, the limitations of this single criterion should not be overlooked. Furthermore, as genetic gain in many subjective quality traits is difficult to determine, quality factors may receive less attention than they merit as breeders concentrate on easily determined yardsticks.

### Product profiles, target population of environments, and benefits

The decision as to what variety or cultivar to plant is usually taken by the farmer. In the private sector, the primary target population is clearly the farmers. Breeders’ objectives are defined with a view to providing farmers with varieties that they desire and will grow. Farmers’ choice of varieties is largely based on the profitability of growing them and how they fit into the farmer’s cropping and management system. Furthermore, in the private sector, those breeders whose varieties are not adopted by farmers simply go out of business and there is automatic selection against the breeding teams who do not understand farming and what drives farmers.

Breeders in the public or quasi-public sector, such as the CGIAR, have a more complex task as their main goals are often determined by the mission of the organizations and donor agencies to which they pertain rather than being a commercial business proposition with farmers as their clients. The donor requirements and the mission of the CGIAR itself go far beyond simply providing farmers with the varieties they desire, often including social benefits for the consumers of farm products and other long-term social and environmental goals (for more details, see ‘Release and adoption of new varieties’, and Data 1 at Zenodo).

One simple approach to developing product profiles was proposed by Cobb *et al.* (2019). An existing variety popular with farmers is identified and the characteristics that make it attractive are appraised by both growers and other value chain players. At the same time, the deficiencies in the variety as perceived by the stakeholders are evaluated. From this, the breeders can determine a list of ‘must-have’ traits and ‘value-added’ traits. Cobb *et al.* (2019) point out that this approach leads to incremental improvement as opposed to creation of the ideal variety, which would take an excessively long time.

When defining the product profiles, the CI programs have to know who is going to grow the crop, where it is to be grown, what are the preferences of both the farmers and the consumers of those crops, who is going to benefit from the adoption of these varieties, and how that adoption will impact on the environment. Although the concept of recommendation domains originally had a strong social component, there was a tendency to treat the people who are to benefit and the environment separately. However, we suggest that people are part of the environment. They interact with the environment and their preferences and circumstances affect what crops farmers grow and how they manage them. Nevertheless, the CGIAR breeders have concentrated on G×E, with E largely determined first by the climate with soils added later. The environments were often classified into large homogenous areas for selection of suitable genotypes (see, for example, Hyman *et al.*, 2013). However, within these homogenous areas or mega environments, the environment varied and the concept of TPE, with some variation of environmental conditions within these mega environments, was developed and has now become the norm (see, for example, Cooper *et al.*, 2021). Breeders were also aware that there are large variations in crop management (M), and the G×E×M interaction should be considered. Furthermore, crop management itself is influenced by the socio-economic circumstances (S). Thus, for example, farmers may not apply fertilizer even though they realize it would be profitable if they have neither available cash nor credit to purchase it. Additionally, the acceptance of a variety will often depend on local preferences by the farmers or the consumers. Hence, the current trend to include socio-economic factors in GEMS and the need to incorporate them in what we suggest should be TPE-S (for more details, see Data 10 and 16 at Zenodo). The additional dimensions, M and S, provide the foundation for more effective design and implementation of pathways to impact.

Despite this relatively well-developed conceptual background of GEMS, we have surprisingly little systematic or representative data on production environments and production practices. Management factors are largely additive (Aune and Bationo, 2008) and farmers may adopt them in a stepwise fashion (Fermont *et al.*, 2009); however, these aspects are not normally recognized in the selection process: there are relatively few data on what farmers actually do in terms of crop management and which practices are related to increasing yields or closing the yield gap (for more details, see Data 11 at Zenodo). There is a clear need for a paradigm shift towards a data-driven approach to identify how farmers manage their crops and how they adopt new practices within their social constraints (for more details, see Data 1, 4, 10, and 12 at Zenodo).

Interpreting the CGIAR mission statement, the CGIAR’s main target populations are currently: (i) those in the world who are not adequately fed; (ii) the smallholder farmers and those that work on the land; and (iii) the poor with emphasis on women and youth. Furthermore, as the poor are concentrated in the rural areas where agriculture is the principal activity, the rural poor are a major target population, with the urban poor targeted through low-cost food that is more nutritious. Tacitly, the CGIAR has another target population that they must satisfy: the multiple agencies that finance the activities of the centers and the higher level decision-makers in the CGIAR system itself. These agencies and decision-makers are not only responsible for financing CI, but also influence the breeding agenda.

Evidently the farmers who make the decision of whether to plant a new variety are the eventual target population of CGIAR breeders, although the materials developed by the centers in pre-breeding may be populations that are used by local programs to produce the varieties that farmers choose to plant. If farmers do not accept and grow a new variety, then the CI program can be deemed a total failure. We note that some breeders in the CGIAR system have contested this point of view, suggesting that if they provide adequate pre-bred materials, they are not accountable for the failure of others to develop varieties that farmers adopt. Most of us do not accept this point of view and believe that CI programs should establish pathways that ensure adoption of the improved varieties. Meeting farmers’ requirements for a new variety is a *sine qua non* for all breeding programs.

While CI programs must ensure that farmers obtain varieties that they will adopt, they are also bound to put materials into the pipeline that not only will satisfy farmers, but will also reach distinct populations defined in the CGIARs mission. This conflict is illustrated by the sad story of *opaque 2* corn. In the mid-1960s, the *opaque 2* gene was discovered in maize (Mertz *et al.*, 1964). This gene was associated with an improved amino acid profile and hence nutritional value of maize. In 1970, at the urgent initiative of the United Nations Development Programme, CIMMYT undertook to improve high-yielding varieties with the incorporation of a gene that promised maize with all the protein quality of milk. However, as noted at the time, CIMMYT faced the more problematic challenge of getting their product into the stomachs of the people who need it (Wolf, 1975). The challenge was never met, the yields were below those of normal maize varieties, and consumers did not like the grain quality: the much-heralded quality protein maize was never widely grown by farmers. Thus, well-meaning donors or policy makers may force breeders to attempt to satisfy conflicting goals with a high probability that they will fail. As discussed above, we can learn from these technical failures when they are adequately understood and accurately documented. The more recent Harvest Plus program had the opportunity to learn from these experiences (for more details, see Data 1 at Zenodo).

In the past 20 years, the CGIAR has once again stressed the importance of nutritional value of crops. The reasons are clear. There is widespread micronutrient undernutrition in low- and middle-income countries (LMICs) (see, for example, UNICEF, WHO and The World Bank Group, 2020), and donors and governments rightly expect the CI interventions to play a role in alleviating this situation (Murray-Kolb *et al.*, 2017; Scott *et al.*, 2018). One of the CI challenges is how to effectively combine the high nutrient densities (or any other additional target trait) with yield and agronomic performance into a single package that can generate profitable varieties for farmers in country crop-specific contexts. This can prove extremely difficult when the genetic control of the trait and physiological make-up are complex and may generate trade-offs. Furthermore, in most of the LMICs, there is no market promotion of nutritionally enhanced products, and few if any incentives for farmers to produce them. In these circumstances, CI teams have to ensure that other important traits are improved so that farmers will adopt the new varieties, while including additional nutrition-related targets. Despite the many hurdles, biofortified products have been produced (e.g. vitamin A-fortified sweet potato, cassava, rice, and bananas, iron- and zinc-rich beans, wheat, and millets, and vitamin A-rich maize) and are being adopted by farmers with measurable benefits to target stakeholders. Thus, for example in Rwanda, clinical trials revealed that the lethal consequences of anemia and iron deficiency could be averted by incorporation of iron-rich beans in the diet of inadequately nourished women (Murray-Kolb *et al.*, 2017).

‘Yield drag’, with the nutritionally improved varieties lagging behind the best agronomic varieties, may impede their adoption (Stone and Glover, 2017). A major feature of the successful cases of improved nutrient status is ensuring that the breeders only make available nutrient-enhanced materials to farmers. It is likely that if some breeders were to concentrate solely on providing varieties with traits that farmers and consumers preferred, these varieties would be preferentially adopted over the new nutrient-enhanced varieties, which would have been subject to trade-offs in their development due to incorporation of a wider range of traits. The successful deployment of nutrient-enhanced crops is a striking example of the importance of combining expertise in many fields and coordinating strategies, in this case releasing only nutritionally improved varieties, with all working towards a common goal (for more details, see Data 13–19 at Zenodo).

The CGIAR stresses the importance of equitable distribution of the benefits that result from its research and that of its partners. Smallholder farmers and other value chain players benefit from better health and nutrition and greater availability of food. Gender is emphasized, with a clear purpose to make the lives of women more agreeable. Gender analysis is central to understanding varietal trait preferences of men and women along the value chain from farmers to final consumers (Data 17 at Zenodo). In South Africa, the crop traits preferred by women who worked on the land differed from those preferred by men (Gouse *et al.*, 2016). Adoption of cassava varieties in Nigeria was strongly associated with traits related to various processes normally carried out by women such as cooking time and ease of peeling (Agwu *et al.*, 2007). Similarly, fast-cooking bean varieties that make life easier for women in Uganda are now being grown by farmers. Thus, understanding the roles that women play in crop production, processing, and marketing should be considered when fixing CI objectives with the possibility of making their life more productive and pleasant.

Farming has shifted from being totally dependent on the availability of natural resources in the farmers’ fields towards creating an, at least partially, artificial man-made environment for crops. This initial modernization of agriculture led to a shift from varieties suited to a specific environment towards modifying the environment to suit the varieties. In this scenario, of modifications to the environment, the immediate solution to the rise of the brown plant hopper, a rice pest, was to modify the environment, in this case with pesticides, to rid fields of pests. By adapting the environment to the crop, single varieties became widespread over large geographical areas and even across continents. Thus, a small number of improved varieties dominated many of the major crops (see Data 12 at Zenodo). Under these circumstances, broad environmental classifications largely based on climate or pest distribution were sufficient for defining most breeders’ target environments. However, there has been a shift back towards molding varieties to the environment. This can be seen by the large number of varieties adapted to local environmental conditions, regionally accessible management practices, and community consumer preferences. To develop varieties for specific local preferences, breeders need to characterize the milieu in which their varieties will be grown, with special reference to the environmental and crop management factors that affect crop performance. It should be noted that the management itself is influenced by the socio-economic setting which will determine the options open to farmers. Furthermore, breeders, who like to see and are often evaluated on the area grown to their varieties, need to know the extent of similar domains before deciding which ones to target.

The old, largely climate-based, generic, classifications were typically broad and continue to be useful for many purposes. However, these broad, general classifications that are appropriate for one crop may not be suitable for another. Thus, for example, soil classified as ideal for flooded rice is likely to be disastrous for avocados. Hence breeders, rather than depending on general classifications, often made for another purpose, require characterizations of the target environment so that they can breed materials appropriate for those circumstances.

One of the most difficult tasks breeders face in the selection process is addressing the S component that frequently requires an intimate understanding of local preferences and customs. This is one of the weakest areas of analyzing target populations, and one of the main reasons for varieties not being adopted (Thiele *et al.*, 2020). Currently, the lack of information on the social influences beyond the farmers control (S) is stark. This is an area where a range of disciplines including, *inter alia*, social scientists, home economists, and anthropologists, can assist breeders (for more details, see Data 15, 17, and 19 at Zenodo).

A positive development in handling the intricacies of GEMS is seen in the availability of the high-resolution spatial datasets of climates, weather, soils, production systems including multiple cropping, crop, and livestock distributions and production variables, and field sizes. The list is long and expanding, with high-resolution, cheap, and frequent-interval satellite imagery, crowdsourcing, and big data approaches now being routinely applied to better characterize GEMS.

### Genetic variation

Once the product profile and the potential beneficiaries have been defined, the breeders have to gain access to the germplasm with relevant diversity in the traits that they are considering. For the CGIAR programs, genebanks are the major source of that diversity. Globally, >1700 genebanks conserve ~7 million accessions (FAO, 2010), with CGIAR genebanks holding ~10% of these materials (Noriega *et al.*, 2019). Much of this germplasm was collected in the last century (Halewood *et al.*, 2012), starting in the 1970s when modern cultivars began replacing traditional landraces selected and shaped over millennia by agricultural communities living in areas where crops had been domesticated (Harlan, 1972). Landraces and wild relatives in existing collections are invaluable sources of unique alleles controlling traits such as nutrient density, abiotic stress tolerance, and disease and pest resistance (McCouch *et al.*, 2020; for more details, see Data 3, 4, and 6 at Zenodo).

A large number of germplasm collections exist. Some of these have been well evaluated, but others have not (for more details, see Data 7 at Zenodo). In the case of common beans (*Phaseolus vulgaris*), the original collection in the 1960s was focused on disease and pest resistances and market classes. Up to now, about two-thirds of the bean collection kept in the CIAT genebank has been evaluated for anthracnose, angular leaf spot, and common bacterial blight, but this figure drops to 18% for drought tolerance and to 7% for low phosphorus tolerance (Hidalgo and Beebe, 1997). Collectors should not be afraid of going beyond the cultivated gene pools, looking for crop wild relatives in extreme environments that may have useful traits especially for combating stresses (e.g. von Wettberg *et al.*, 2018). Breeders tend to only ‘reach back’ to genebank materials if there is insufficient genetic variation for a trait in elite gene pools since linkage drag and genetic background effects both reduce chances of success in wide crosses (for more details, see Data 3 at Zenodo). This problem is less marked in crops that have only recently been the subject of intensive selective breeding. Additionally, marker-assisted selection or gene editing make it possible to rapidly recover the trait of interest into elite germplasm, especially when the trait is controlled by few genes (Assefa *et al.*, 2019). Candidate genes involved in physiological reactions to stresses such as drought or heat will probably be found in wild species from desert habitats that have for millions of years passed the test of time (Data 7 at Zenodo). We respectfully suggest that searching in these extreme environments may be equally or more enlightening than delving ever deeper into the functionality of genes from Arabidopsis. Recently heat tolerance has been introduced to common beans from wild tepary beans, and several lines in CIAT are now in advanced stages of selection (Souter *et al.*, 2017). Some of these traits such as drought tolerance may be controlled by many genes: in such cases, genomic selection may lead to more rapid introduction of the desired traits to elite germplasm and faster elimination of undesirable traits. There is a certain urgency to prospect these extremes as much of the variation is rapidly disappearing (Data 7 and 8 at Zenodo). Large databases on climate and soils and automated software pipelines to analyze them can now help collectors identify the ecologies where these extreme behaviors are likely to be found (see, for example, Fick and Hijmans, 2017).

Genomics is not only changing breeding systems, but it also opens the way to rapidly identify potential sources of genetic variation: molecular markers can be linked to traits of interest. High-density genotyping of entire collections is now entirely feasible, with costs ranging from less than the cost of a year to a few years of conserving a collection (Data 8 at Zenodo). Breeders need to be able to associate the phenotype with the genotype so that they can develop materials with the required levels of expression of a specific trait in the phenotypes. Technological advances since the early 2000s have facilitated this process with rapid high-throughput sensor-based phenotyping (HTP). This technology, now often labeled, as ‘phenomics’, has the capacity to provide information on specific traits or environmental response curves of hundreds to thousands of genotypes (Tardieu *et al.*, 2018). Phenomics tools can assist in accurate, non-destructive, automated, standardized, cost-effective exploration of plant phenotypes. This approach will be largely limited only by the capacity of researchers to formulate hypotheses and quantitatively define, defend, and justify the tangible phenotyping targets for use in CI and also the ability of HTP systems to mimic the target environment. In addition, given the diversity of crops, cropping systems, and growing conditions, the development of standard data analytical tools for phenotyping will be challenging. Currently, automated or semi-automated methods are being developed with the capacity to evaluate crop phenotypes.

The value of accessible germplasm for breeders is greatly increased if it is accompanied by information on that germplasm. Crop Ontology (CO; Data 9 at Zenodo) is the only ontology that offers a comprehensive list of defined traits along with their pre-composed variables, ready to use in field books or lab books. User-friendly and open-access databases are extremely important if the objective is to effectively utilize the germplasm conserved in the genebanks as sources of diversity for CI. The new, so-called ‘Future Seeds’ genetic resources center being built at the CGIAR hub in Colombia illustrates the future with its goal to gradually assemble, for each crop conserved, a knowledge base that documents: (i) *ex situ* (compared with *in situ*) diversity; (ii) environmental adaptation of accessions; (iii) traits of interest for CI (whether measured or predicted); and (iv) the allelic composition of accessions for genes with known function with a particular focus on functional single nucleotide polymorphisms (SNPs) that could be potential targets for future gene-editing attempts.

A major problem faced by breeders is incorporating traits from unimproved sources into elite germplasm. Pre-breeding attempts to obviate this difficulty by producing germplasm in which desirable traits have been incorporated from unselected germplasm or wild species, and the undesirable traits have been removed. More proactive and systematic pre-breeding efforts could ‘de-risk’ the use of novel genetic variation from genebanks. Core collections (or other succession subsets) could be systematically ‘reformatted’ into ‘bridging germplasm’ through crossing with elite germplasm, as was the case of sorghum populations developed at the University of Queensland that were used to develop widely adopted varieties (Jordan *et al.*, 2011). In the CGIAR system, this type of elite, pre-bred germplasm can be distributed to national and local breeding programs that can then incorporate locally important traits reflecting the preferences of the target community and provide varieties adapted to the specific local environment. This pre-breeding, when carried out in consultation and with the collaboration of breeders, can complement their work and should not be considered as competing with their efforts.

### Diseases and pests

Although disease or pest resistance is rarely the main target of CI, resistance to specific diseases and pests is an integral part of most CI programs. However, host plant resistance is not necessarily the optimum strategy for managing a specific disease or pest. Expertise in crop protection, provided by, amongst others, plant pathologists and entomologists, plays an important part in deciding if a disease or pest is sufficiently severe to merit attention and, if so, whether host plant resistance is the most viable method of control. When host plant resistance is included in the product profile, the pathologists and entomologists can provide information on the genetic variability in the trait, the nature of the resistance or tolerance, and how rapidly and easily it can be evaluated and incorporated into the breeding populations.

Host plant resistance is often first discovered in genotypes that do not have desirable agronomic traits. This is most marked when resistance is found in another species. Introduction of resistance from less desirable types into elite improved populations is a major challenge (see earlier). Technologies including transgenesis, marker-assisted selection, and genomic selection can facilitate introduction of resistant genes into elite lines.

Currently, resistance is most frequently evaluated by visual scoring and subjective methods that were not developed for capturing minor differences between genotypes, which are the basis for quantitative, durable, resistance. Investment is needed for the development of HTP which will accelerate the move from marker-assisted selection to more robust genomic selection.

One of the consequences of the Red Queen effect (see earlier) is that breeders should, whenever possible, be aware of potential threats and have germplasm ready to combat them when they occur. This may require testing or screening populations for resistance to diseases and pests in areas where they are present, even though they are not currently a problem in the target area. Marker-assisted and genome-wide assisted selection may reduce the need to physically move germplasm to screen for resistance, thus obviating problems of quarantine and phytosanitary restrictions on moving plant materials and their pathogens or pests.

Although breeding objectives should not be frequently changed, pathologists and entomologists should periodically review the status of plant resistance to a particular disease or pest, or the appearance of a previously unknown disease or unreported disease in the target environment. This information can then be used to modify breeding objectives or product profiles.

### Crop productivity

Yield, or productivity per unit land area, is usually amongst the most important breeding goals. Although breeders will be looking for genetic gain in a series of traits, they will always focus on yield: *reductio ad absurdum* if there is no yield, there is no crop! However, breeders should not lose sight of the farmers’ goals which are not yield *per se.* Farmers are, we suggest, interested in improving their livelihoods, and this may be more related to increasing their incomes and reducing drudgery rather than simply increasing yields. Furthermore, farmers may prefer a lower yielding variety that, due to inherent quality characteristics, commands a premium price in the local market. Hence, CI programs should be aware of the dangers of a single-minded focus on yield. Within this line of thought, for farmers and those that work on farms, labor productivity may be of equal or more importance than simply producing more.

#### Yield

Yield is often taken as a character on its own. However, it is far too complex to be considered as a single trait. From a CI point of view, there are various ways of looking at yield. In the past, large yield increases were common; however, the yield increase of food crops is likely to decrease from a current value of 1.2%/year to 0.66%/year by 2040 and to 0.50%/year 2050 (Fischer and Connor, 2018; see Data 11 at Zenodo). In any location, the potential yield (PY) of any crop is the yield obtainable with the most adapted cultivar grown under the best management practices, with no biotic stress. This yield will be more variable under rainfed conditions (PYw) than under irrigation (PY) due to year to year variation in rainfall. Best practice agronomy is not static but develops with time, and farmers commonly identify an economic yield which is 20–30% less than PY (Grassini *et al.*, 2011; Fischer *et al.*, 2014). However, PY remains an important benchmark because it can be more closely achieved if prices for products increase.

The difference between PY and what farmers achieve (FY) establishes a yield gap (Yg=PY–FY or PYw–FY) that defines the yield gain that is possible with current cultivars. Breeding seeks to increase PY while agronomic practice seeks to increase FY and reduce Yg. There are large differences in Yg between crops. In the major starch staples, Yg is smallest in wheat and largest in cassava (see Data 11 at Zenodo). In general terms, when Yg is small, greater PY can only be obtained from genetic gain. When Yg is large, a faster and surer route to greater FY is through improved agronomic practice and attention to social and economic factors that limit adoption by farmers.

For the breeders, a knowledge of how individual traits can be combined to obtain increased yield under specific conditions is invaluable. For example, an understanding of photoperiod effects on many crops, including rice, wheat, and soybeans, led to the development of photoperiod-insensitive varieties which are now the norm. Similarly, stomatal sensitivity to air humidity, first reported by Schulze *et al.* (1972) and later suggested as a major feature of drought resistance in cassava (see, for example, El-Sharkawy *et al.*, 1984), contributes to drought tolerance in maize varieties that have become popular in the US corn belt (Messina *et al.*, 2015). Thus, an understanding of the crop’s response to the environment and management can help breeders select for individual traits which will enhance yield under specific conditions.

A major concern for breeders is how incorporating a single trait, hopefully associated with increased yield, will interact with other traits. Thus, for example, a breeder may wish to know whether breeding for erect leaves, often associated with high yields of some crops in the dry season, is a good strategy for crops grown in the cloudy wet season. Design of experiments to sort out this conundrum is extremely difficult. However, crop simulation models offer the possibility of rapidly solving such riddles by transiting from decisions based on results from experiments *in vivo* to the use of predictions based on cropping systems simulation *in silico*. Moreover, crop modeling is the sole approach that can comprehensively evaluate crop response to the future environments anticipated with anthropogenic climate change (Harrison *et al.*, 2014; Hammer *et al.*, 2020). Crop models that encapsulate how plants respond to dynamic variation in the environment are increasingly being used in GEMS characterization. For example, simulations can enable detailed quantification of the soil–crop water status dynamics through the life cycle of a crop over a range of likely variations in the weather patterns for a particular target environment (Messina *et al.*, 2011; Cooper *et al.*, 2014; Xu, 2016; Bustos-Korts *et al.*, 2019). Hence, we suggest that more emphasis should be placed on developing models that can *in silico* test new hypotheses and provide breeders with greater certainty of the likely results of combining distinct traits. This becomes of great importance when a particular trait may lead to improved performance under one set of conditions, and less under another.

When considering yield, breeders pay attention not only to traits that can enhance yield under specific conditions, such as the example given above for tolerance of dry conditions, but also to minimize biotic factors such as diseases and pests that reduce yield with emphasis on host plant resistance.

#### Labor productivity

The current importance of rural poverty alleviation, preferably stated as improved welfare and prosperity, suggests a need for improved labor productivity in agriculture. The labor productivity gap in agriculture between the developing and the developed world is frequently greater than an order of magnitude (see, for example, Gollin *et al.*, 2014). This gap is greater than that of crop yields. In a globalized world in which developing country producers compete with those from the developed world, it is impossible to pay those who work on the farm a reasonable wage unless labor productivity is increased. Despite this situation, the CGIAR CI programs rarely consider traits that improve labor productivity in their product profiles: this gap should in the future be filled (for more details, see Data 1 at Zenodo).

The inclusion of robots in agriculture is likely to have a major impact on both the precision of many field operations and the labor productivity. In precisely the same manner that breeders have developed crops suitable for mechanization, food processing, and industrial use, in the future they will surely have to develop crop varieties that facilitate the use of robots.

#### Total factor productivity

Many farmers in developing countries have limited capital to invest, and hence capital productivity is also important. This is likely to be a major constraint to increasing incomes and reducing rural poverty through the production of high-value crops.

#### Quality

One of the simplest ways for a farmer to increase his/her productivity, measured not in terms of yield but rather in terms of economic gain, is to obtain a price premium due to the inherent quality of the product. CI programs should not lose sight of the importance of product quality as a means to increase economic yield.

## Conclusions

The world is changing rapidly with, *inter alia*, new technology becoming available, society everyday better educated, and with the expectation of a better life. All this occurs as we move into the Anthropocene age with large changes in the climate and weather patterns which are crucial for agriculture. The conclusions drawn here should be appraised within this framework of a rapidly changing global scenario which requires agile and opportune responses.

- The CGIAR’s mission has evolved from principally increasing the production of staple crops to feed the world to meeting multiple social and environmental goals.
- The primary goal of breeders in the private sector is to provide farmers with varieties they will adopt, whereas those in the CGIAR are charged with achieving multiple physical, biological, environmental, and social goals.
- The CGIAR should review its portfolio of crops to ensure that it is commensurate with the scope of its constantly evolving mission and goals.
- As One CGIAR consolidates, it is likely that distinct crops in its portfolio will play distinct roles in meeting the varied goals. Thus, some crops may be destined to play a major part in creating rural prosperity and a more equitable society, and others for providing low-cost nutritious food. The CGIAR should provide CI programs with guidance on the role their crop is expected to play.
- While CGIAR breeders are charged with major impacts on society at large by including such traits as improved nutritional value, it is the farmers who decide whether to grow them or not. Hence, CI programs must balance the needs for society at large or the market for farm produce with the need to ensure that farmers prefer and adopt the improved varieties.
- Clear goals and closely knit interdisciplinary teams have been key features of successful CI in the CGIAR. Hence, clear breeding objectives and product profiles and a range of expertise in crops are essential ingredients of CI programs.
- The private sector uses the concept of product profiles to determine breeding objectives. Product profiles should be more broadly adopted by the CGIAR programs with special reference to ensuring the balance between farmers needs and those of society at large. This may lead to a shift away from the traditional, primary objective of increased yield of staple crops.
- The inclusion of social objectives in the breeding agenda may cause ‘yield drag’ which may inhibit adoption of varieties by farmers. Breeders and policy makers need to collaborate to ensure that yield drag does not prevent the adoption of socially advantageous varieties.
- The days when a single variety was grown across large swathes of the world are gone. The trend is towards varieties specifically adapted to local conditions and preferences, with increasingly myriad varieties selected to meet local conditions and preferences.
- Recommendation domains, that consider relatively homogenous realms with similar conditions, originally developed in the CGIAR system included social aspects. Later they became largely biophysical with emphasis on the G×E. The more holistic inclusion of both the management (M) and society (S) components in both TPE and GEMS is seen as a massive step forward. The conceptual base of TPE with a greater social and management component (TPE-S) and GEMS is evolving rapidly, with people or society increasingly seen as an integral part of the environment. However, social and crop management aspects are still weak and need to be improved. The rapidly evolving methods and tools to characterize TPE-S and evaluate GEMS should be embedded in the core CI teams.
- Pre-breeding is used by many CGIAR CI programs to provide international and national programs with elite materials that can be used to develop varieties that meet local requirements and preferences. In this process, the CGIAR not only must provide genetic material but also must strengthen local capacity to deliver new varieties to farmers. It is not acceptable for pre-breeders to lament if national or local programs do not deliver. Within this context, the maintenance of fruitful relationships between the consultative group as an international development-oriented research organization and partners, including academic institutions and national research and development organizations, is crucial.
- The importance of germplasm banks as a source of genetic variation is evident. Their utility depends on their accessibility and characterization. Currently there are many new methodologies and tools available to characterize them and to make this information available. The CGIAR should invest more in the characterization and evaluation of the conserved germplasm in order to make more materials and information available to the breeders and pre-breeders. This may require the establishment of new facilities for characterization.
- CI programs should be wary of short cuts and silver bullets. It is essential to understand the whole system and the interactions between the distinct alleles and corresponding traits that are combined.
- Modern data collection, digitalization, and information management systems are revolutionizing CI. These run from high-throughput phenotyping, GIS, genomic-wide association selection, meteorology, and soil characterization through to monitoring of farm management practices, including the performance of cultivarss and the social ambience.
- *In silico* methodologies are becoming increasingly important to evaluate possible combinations of alleles and traits and their performance over a wide range of conditions, often replacing expensive field trials. However, there are dangers of sitting in front of a computer in an artificial world and becoming divorced from reality.
- Contact with farmers and monitoring of what is happening in the real world, especially on farms, is essential and the CGIAR should strengthen its capacity in this area.
- Genetic gain is an intellectually attractive yardstick, but dangerous if not used well. If genetic gain is used to measure a breeder’s success, breeders are likely to concentrate on those traits for which genetic gain is readily measured, while missing those that are difficult to quantify, such as maintenance breeding.
- Breeders will encounter failures in their endeavors. They must learn from these experiences.
- CI is a long-term venture with continuity an essential ingredient. The current short-term project structure of the CGIAR is not appropriate. The ‘call for proposal syndrome’ as it is commonly known should be avoided in CGIAR system. Wherever possible, longer term ‘programmatic’ financing should be preferred over short-term ‘project-oriented’ financing. The return to longer term program financing should be coupled with continual monitoring and review of programmatic progress.
- Breeders should not be overambitious using CI as a tool to resolve many problems. They should resist pressure particularly from donors to do the impossible!
- Although some countries do not accept GMOs and GEds, the CGIAR should provide them to those countries that approve of them, whilst taking every precaution against misuse.

## Data Availability

Further data are available at Zenodo: https://doi.org/10.5281/zenodo.4638248; Kholová *et al.* (2021).
